# Author’s Response

**DOI:** 10.1308/003588412X13373405386330

**Published:** 2012-09

**Authors:** Jonathan Weir-McCall

We thank the authors for their interest in our article and are interested to hear that our findings correlate well with that of their own experience. We did not intend to discount the effect of clinical judgement and indeed the study was not designed to assess this, as it did not include patients who never required CT, or those who had CTs but never required operative intervention.

The accuracy of clinical judgement and the effects of CT on this have been well documented elsewhere.^1^ The purpose of the study was to quantify the degree of confidence surgeons could place in the CT findings when they are considering operative intervention. The role of CT being an adjunct rather than the decisive factor in decision making was well demonstrated in our study as 11% of patients with inaccurate reports progressed to have a non-exploratory operative intervention. Our study showed that only 3% of patients had a negative laparotomy, which is surely the best indicator that by working as a team surgeons and radiologists can come to a very accurate decision of when operative intervention is necessary.
